# Three Mexican Families with β thalassemia intermedia with different molecular basis

**DOI:** 10.1590/1678-4685-GMB-2019-0032

**Published:** 2020-02-03

**Authors:** Lourdes del Carmen Rizo de la Torre, Francisco Javier Perea Díaz, Bertha Ibarra Cortés, Víctor Manuel Rentería López, Josefina Yoaly Sánchez López, Francisco Javier Sánchez Anzaldo, María Teresa Magaña Torres, Katia Gonnet, Catherine Badens, Nathalie Bonello-Palot

**Affiliations:** 1 Instituto Mexicano del Seguro Social, Centro de Investigación Biomédica de Occidente, División de Medicina Molecular, Independencia Oriente, Guadalajara Jalisco, México.; 2 Instituto Mexicano del Seguro Social, Centro de Investigación Biomédica de Occidente, División de Genética, Independencia Oriente, Guadalajara Jalisco, México.; 3 Universidad de Guadalajara, Centro Universitario de Ciencias de la Salud, Instituto de Genética Humana “Dr Enrique Corona Rivera”, Independencia Oriente, Guadalajara Jalisco, México.; 4 Laboratorios Ruiz, Puebla de Zaragoza, Puebla, México.; 5 Hôpital d’ enfants de la Timone, Déparement de Génétique, Laboratoire de Génétique Moléculaire, Marseille, France.

**Keywords:** Thalassemia intermedia, Mexican population, β globin gene, new mutations, alpha-globin gene duplication

## Abstract

Beta thalassemia (β-thal) is a frequent monogenic disease, is clinically and molecularly heterogeneous. This study described molecular and laboratory findings for three Mexican patients with β-thal intermedia phenotype and their relatives. Three Mexican families were studied for presenting β-thal intermedia, ARMS-PCR and Gap-PCR were performed to screen for common mutations, Sanger sequencing for rare or new alleles, and MLPA for identifying deletions and or duplications. In all three families we observed, in heterozygote condition, the mutation c.118C > T (p.Gln39*) also known as codon 39(C > T) in the β globin gene (*HBB*) associated with a novel molecular defect: a new duplication of the alpha globin gene cluster, a new deletion that includes the loss of exon 3 of HBB and finally a novel mutation in the 3’UTR of HBB (*HBB*: c.*132C > A). We report three Mexican families with beta thalassemia intermedia due to different molecular basis; a new single nucleotide mutation involving the last nucleotide of the β-globin chain transcript; and two possible new DNA rearrangements, an α cluster duplication, and a partial β gene deletion.

## Introduction

Beta thalassemia (β-thal) is one of the most frequent worldwide monogenic diseases, it is an autosomal recessive disorder, characterized by reduced (β^+^, β^++^) or absence (β^0^) of hemoglobin’s beta subunit (Thein, 2018). *HBB* gene mutations are the cause for this disease, to date there are near 300 reported β-thal alleles (http://globin.cse.psu.edu/), these mutations can be divided in three main groups: a) transcriptional mutations that include promoter regulatory elements or 5’ UTR; b) RNA processing mutations involving splicing junction, consensus and cryptic sequences, poly A signal and 3’ UTR; and c) translation mutations which include initiation codon substitutions, nonsense and frameshift mutations (Thein, 2018). Although single nucleotide mutations represent the vast majority of β-thalassemias, deletions involving *HBB* gene, or its regulating sequences have also been associated to this pathology (Patrinos GP *et al.*, 2004). Despite being an autosomal recessive inherited condition, some forms of β-thal are inherited dominantly (β^D^ alleles), so is the case of nonsense or frameshift mutations in exon three that result in the formation of a premature termination codon and therefore the production of unstable β-globin chains that precipitate along with the free α-globin chains in the erythroid precursor causing premature death and ineffective erythropoiesis (Thein, 2018; Rawa *et al.*, 2017).

β-thal is classified according to severity as minor, major and intermedia (Thein and Wood *et al.*, 2009; Thein, 2018). The minor form corresponds to the simple heterozygote condition whereas the beta-thalassemia major type refers to the more severe disorder with transfusion dependency as a result of the inheritance of two β^0^ alleles. β-thal intermedia comprises a wide range of clinical features and is mainly characterized by occasional blood transfusions; β-thal intermedia is observed in different molecular forms such as, two β-thal alleles (β^0^/β^+/+^, β^0^/β^0^), one β-thal allele and additional copies of α-globin genes (β^0^/β^A^; ααα/αα, αααα/αα, αααα/ααα) or the presence of a single dominant β-thal allele (β^D^/β^A^) (Origa *et al.*, 2014; Ben-Salah *et al.*, 2017, Clark *et al.*, 2018).

The pathophysiology of this disease is the outcome of an imbalanced ratio of α- and non-α-globin chains (excess of α-globin chains), and the severity depends on three different mechanisms. First, the type of β allele (β^0^, β^+^ or β^++^), correlated to the amount of residual β-globin chains (Mettananda *et al.*, 2015; Ben-Salah *et al.*, 2017; Shang and Xu 2017; Zhong *et al.*, 2018). Second is the co-inheritance of alpha globin locus defect, either an alpha-thalassemia deletion (α-thal) which is often observed in β-thal patients, resulting as a positive modifier or, conversely, the presence of additional copies of *HBA2* or *HBA1* genes which causes a more severe form of β-thal (Ben-Salah *et al.*, 2017; Theodoridou *et al.*, 2018; Clark *et al.*, 2018; Harteveld *et al.*, 2008). And third is the presence of genetic variants associated with increased fetal hemoglobin (HbF) production. For instance, *HBG2*:c.-211C > T (*Xmn*1 -158C > T) a single nucleotide polymorphism in the *HBG2* promoter is known to be a HbF inducer (Mettananda and Higgs 2018; Ngo and Steinberg 2015; Sripichai and Fucharoen 2016).

Beta thalassemic alleles have been previously studied in Mexican population, a total of 21 different mutations represent the molecular spectrum of this disease in Mexico (Rizo-de la Torre *et al.*, 2017). The aim of this paper is to report for the first time three Mexican families with β-thalassemia intermedia due to the combination of the common allele (*HBB*: c.118C > T) and a novel molecular defect.

## Subjects and Methods

### Subjects

Three thalassemia intermedia patients and their families were included in this study.

Family 1 is composed by seven members originally from the state of Jalisco, the index case is a 25-year-old female. Family 2 is from Oaxaca and consists of a 5-year-old girl and her parents. Family 3 is from Guanajuato and composed of three members, a 28-year-old male and his two children.

All subjects signed informed consent for hematological, biochemical and molecular studies. All procedures were performed in accordance with the standards of the local ethics committee of the Instituto Mexicano del Seguro Social (Mexican Institute of Social Security) and with the 1964 Helsinki declaration.

#### Hematological and biochemical analysis

Peripheral blood (10 mL) was collected in EDTA tubes. The hematological data were obtained by electronic counter (ADVIA2120i; Siemens Healthcare, Erlangen, Germany); the biochemical analysis was performed by conventional methods (HbF by alkaline denaturation, HbA_2_ by microchromatography with diethylaminoethyl DEAE – cellulose–Sigma Aldrich and, electrophoresis in cellulose acetate) (Huisman and Jonxis, 1977).

### Molecular analysis

DNA samples were isolated by salting-out procedure (Miller *et al.*, 1988). The genotyping process comprises the routine thalassemia screening: ARMS-PCR for *HBB* known mutations (Old *et al.*, 1990), gap-PCR for δβ-thalassemia Spanish type (Vives-Corrons *et al.*, 1992) and common alpha-thalassemia deletions (Liu *et al.*, 2000). Furthermore, *HBB*, *HBA2* and *HBA1* genes were analyzed by Sanger sequencing, using BigDye® Terminator kit v3.1 and Applied Biosystems 3500 Series Genetic Analyzer (Applied Biosystems); and multiple ligation-dependent probe amplification using SALSA® MLPA® probemix P102HBB and P140HBA following the manufacturer’s recommendations (MRC-Holland) for all the individuals described above.

## Results

All three propositus were suspected of intermedia thalassemia. They exhibited mild to severe anemia, and unusual elevated HbF. We suspected the presence of at least two thalassemic alleles (α, β or δ), and/or hereditary persistence of fetal hemoglobin. Hematological, biochemical and molecular data are shown in Table 1.

**Table 1:** Hematological, biochemical and molecular data of three Mexican families with β-thal intermedia.

Subject	Gender/Age	RBC (10^12^/L)	Hb (g/dL)	MCV (fL)	MCH (pg)	HbA_2_ (%)	HbF (%)	β Genotype	α Genotype	γ Genotype*
Family 1										
I-1^§^	M/48	3.0	10.5	100.0	33.3	1.8	1.6	β^A^/β^A^	αααα/αα	C/C
I-2	F/46	5.1	10.6	65.5	20.8	3.3	2.2	β^39^/β^A^	αα/αα	C/T
II-1^† (II-1†§)^	F/33	1.8	4.8	85.1	26.8	3.8	11.7	β^39^/β^A^	αααα/αα	C/C
II-2	F/32	5.3	13.9	80.3	26.1	1.1	1.7	β^A^/β^A^	αααα/αα	C/T
**II-3**	**F/25**	**3.1**	**6.2**	**66.1**	**20.3**	**3.8**	**6.7**	β^39^/β^A^	αααα/αα	**C/C**
II-4	F/21	3.3	7.4	70.9	22.4	2.1	17.9	β^39^/β^A^	αααα/αα	C/C
II-5	M/19	4.6	14.7	93.2	32.1	2.2	1.6	β^A^/β^A^	αα/αα	C/T
Family 2										
I-1	M/25	6.1	12.3	65.7	20.3	5.2	1.4	β^39^/β^A^	αα/αα	C/C
I-2	F/25	5.3	10.2	65.0	19.1	4.9	6.7	β^del^/β^A^	αα/αα	C/T
**II-1**	**F/5**	**3.8**	**9.8**	**88.3**	**26.1**	**0.5**	**80.5**	β^39^/β^del^	αα/αα	**C/T**
Family 3										
**I-1**	**M/28**	**3.6**	**7.1**	**82.0**	**19.9**	**7.8**	**8.7**	β^39^/β^+132^	αα/αα	**C/C**
II-1	F/7	6.2	11.8	58.0	19.1	6.3	3.4	β^39^/β^A^	αα/αα	C/C
II-2	M/1	4.2	10.7	76.7	25.2	3.6	8.4	β^A^/β^+132^	αα/αα	C/C

RBC=red blood cells; Hb=hemoglobin; MCV=mean corpuscular volume; MCH=mean corpuscular hemoglobin; HbA_2_=hemoglobin A_2_; HbF=fetal hemoglobin; β^39^=*HBB*:c.118C > T; β^del^=*HBB* 3’ deletion; β^+132^=*HBB*:c.*132C > A. Index cases are shown in bold. * = *HBG2*:c.-211C > T (*Xmn*1 -158C > T). † Transfused during sample collection. § Deceased after diagnosis.

Family 1

The proposita (II-3) and her two sisters (II-1 and II-4) presented a profile typical of thalassemia intermedia (Table 1) whereas the phenotype of her parents does not entirely resemble a β-thal carrier, despite both of them presented mild anemia. Microcytosis and hypochromia were only observed in her mother (I-2), who also had borderline HbA_2_. Her father presented macrocytosis, low HbA_2_ and normal HbF.

**Figure 1:**
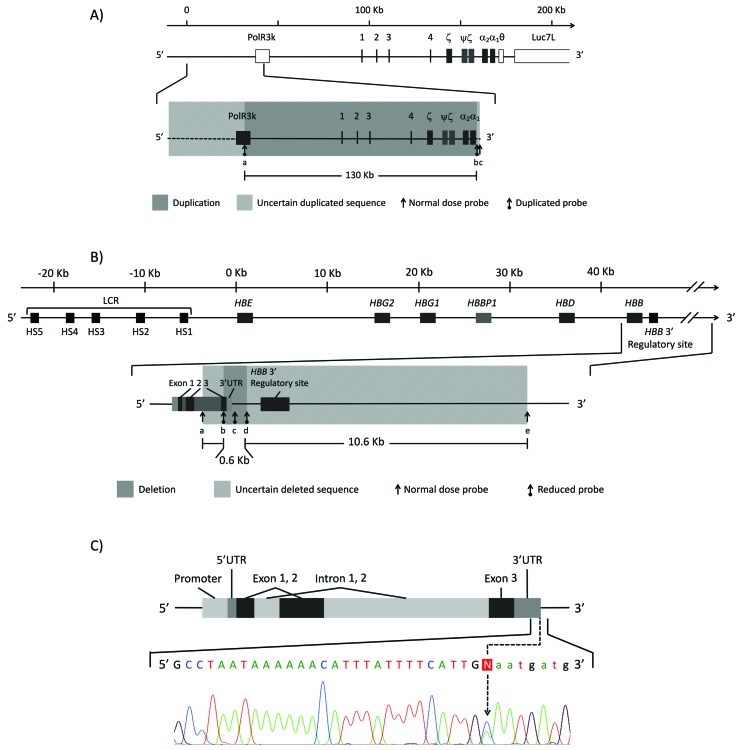
Schematic representation of three globin gene mutations associated with *HBB*.c:118C > T in patients with β-thal intermedia. A) α-cluster duplication from Family 1. Duplication is shown in dark grey shade, uncertain breakpoints are indicated in light grey shade; MCS-R sequences are numbered from 1-4; involved MLPA probes are specified by (a) 463 bp 19236-L25316, (b) 283 bp 04638-L23602, and (c) 310 bp 04639-L04020. B) *HBB* 3’ deletion from Family 2. Deletion is indicated in dark grey shade, uncertain breakpoints are shown in light grey shade involved MLPA probes are presented by (a) 196 bp 05833-L05335, (b), 166 bp 11884-L12684 (c) 206 bp 11885-L13080, (d) 173 bp 05836-L06321; (e) 274 bp 11980-L12803. C) HBB:c.*132C > A point mutation found in Family 3. Nucleotide change is indicated with an arrow. Nucleotides in capital letters represent transcribing sequence.

We found by Sanger sequencing the mutation *HBB*: c.118C > T (p.Gln39*) (or β39C > T) in heterozygous condition in her mother (I-2) and the three affected members (II-1, II-3 and II-4). The second molecular defect observed in the three affected patients was a large duplication in α cluster that includes the regulatory HS-40 region as well as the genes *HBZ*, *HBPZ*, *HBM*, *HBPA1*, *HBA2* and *HBA1:* 16p13.3 (?-037132)_(167890-169744) (Figure 1A). This molecular defect was inherited from her father and transmitted to 4 members of the family (3 affected (II-1, II-3 and II-4) and one unaffected (II-2)). II-2 presented subnormal MCV and MCH (Table 1, Figure 1A).

Family 2

β Thalassemia intermedia phenotype was observed in the 5-year-old proposita (II-1) with a HbF value remarkably increased (80.5%). By analyzing independent phenotypes of both parents we observed a classical β^0^ allele carrier phenotype in the father (I-1) and her mother (I-2).

By Sanger sequencing of *HBB* gene in the proband, we observed the mutation *HBB*: c.118C > T (p.Gln39*) (or β39C > T) inherited from her father. The second molecular defect identified in the index case, inherited by her mother, was a deletion of the 3’ end of the *HBB* gene, comprising the third exon and the 3’ UTR of *HBB*. This deletion comprises the loss of a segment between 0.6 and 10.6 Kb: 11p15.4 (5193650-5202711)_(5203314-5204229) (Figure 1B). Therefore the β^39C > T^/β^del^ genotype explains the thalassemia intermedia phenotype. Both parents were simple heterozygotes, the father β^39C > T^/β^A^, and the mother β^del^/β^A^.

Family 3

The propositus is a 28-year-old male patient with β-thal intermedia phenotype, elevated HbF (8.7%) as well, and an unusual increment of HbA_2_ (7.8%). His 7-year-old daughter presented a typical β^0^ thalassemia carrier phenotype, with considerably elevated RBC and increased HbA_2_ (6.3%). Moreover, his one-year-old son presented a mild anemia (10.7 g/dL) with elevated HbF (8.4%), with microcytosis and slightly increased HbA_2_ (3.6%).

The molecular analysis by Sanger sequencing of the *HBB* gene revealed the presence of two single nucleotide mutations in compound heterozygosity condition in the propositus: c.118C > T (p.Gln39*) (or β39C > T) and c.*132C > A (Figure 1C). The c.118C > T mutation was transmitted to his daughter (II-1) and the other mutation, c.*132C > A, to his son (II-2).

## Discussion

Beta thalassemia is one of the most commonly studied genetic diseases. It is mainly attributed to single nucleotide mutations in the *HBB* gene, however, association with other globin gene mutations such as *HBA2* and *HBA1* triplication is often observed. In Mexican individuals, a previous report observed the presence of -α^3.7^ and ααα^anti3.7^ in 16% and 28% respectively of β-thal carriers (Nava, *et al.*, 2006). The pathophysiology of β-thal is related to the unbalanced ratio of α/β-globin chains caused by the reduced (β^+^), or absent (β^0^) β-globin synthesis and the relative excess of free α-globin chains. In this paper we report three Mexican families with beta thalassemia intermedia due to different molecular defects.

In the first family, we detected the presence of a single β^0^ allele (*HBB*:c.118C > T) in the proposita and two sisters, whose phenotype resembles thalassemia intermedia since they all have severe but non-transfusion dependent anemia and elevated HbF. In five members of the family (I-1, II-1, II-2, II-3 and II-4), we observed the presence of an α-globin genes duplication encompassing the regulatory HS-40 region. Their hematological data were consistent with previous reports of families carrying one β^0^-thal allele along with an α-cluster duplication (Harteveld et al., 2008; Ben-Salah et al., 2017; Clark et al., 2018; Theodoridou et al., 2018). It is known that co-inheritance of α deletions represents a benefit for β-thal patients; on the other hand, increased α-globin chains due to genes duplication in β-thal patients leads to a more severe phenotype (Mettananda et al., 2015; Saleh-Gohari et al., 2015; Shang and Xu 2017). Several studies demonstrate the common association of α-globin gene duplications and a single β-thal allele as a cause of β-thal intermedia (Origa *et al.*, 2014). To date, there are ten genetic rearrangements that end in additional copies of one or both alpha-globin genes including the worldwide-distributed 3.7 kb and 4.2 kb triplications (Clark *et al.*, 2018). The duplication reported in this study is larger than duplicated segments previously reported. Additional molecular and/or cytogenetic testing such as a Comparative Genome Hybridization could help to understand the origins and exact location of these genetic variants.

The second family presented also thalassemia intermedia phenotype, however, their molecular basis included two β globin gene mutations: the typical *HBB*.c:118C > T and a new uncharacterized deletion involving the 3’ end of *HBB* gene. This deletion causes the loss of the third exon, therefore may produce a short unstable mRNA that could undergo early degradation. This early degradation mechanism is typical for mutations inducing a premature termination codon in coding sequences (Grosso *et al.*, 2008; Rawa *et al.*, 2017). It is important to notice the unusual increased HbF in the mother, who is a simple heterozygote for this deletion. Aside the high HbF level she presents a classical β^0^ thal allele carrier phenotype. There is a large number of deletions in the β-cluster that are associated with elevated HbF, nevertheless, these deletions usually involve the complete loss of gamma, delta and/or beta genes (Andersson et al., 2007; Thein and Wood, 2009; Pissard et al., 2013). The particular deletion reported in this study causes the loss of third exon and the possible loss of the *HBB* 3’ regulatory region (DNase I hypersensitive site; LOC110006319; Gene ID: 110006319), which includes multiple GATA1 binding protein sequences (https://www.ncbi.nlm.nih.gov/gene/107133510). Here again, further research is required to determine the exact breakpoints.

Although it is known that the loss of the 3’ regulatory sequences in *HBB* does not alter its own expression, little is known about its relationship with adult *HBG2* and *HBG1* expression (Bender *et al.*, 2006; Reading *et al.*, 2016). Several deletions concerning the β-globin gene cluster have been reported (Mettananda and Higgs, 2018; Thein 2013). They are classified in three main groups: those that remove the LCR (Locus Controlling Region); others that remove partially or totally the β-cluster genes (∊γδβ^0^-thalassemia, γδβ^0^-thalassemia, δβ^0^-thalassemia); and deletions that involve partial or complete loss of the β-gene, such as, the Asian/Indian 619 bp deletion of *HBB* 3’ end, the ≈45 kb Filipino deletion that removes the entire β-gene, and the recently reported 5’ β-globin gene deletions of 538 bp and 1517 bp (Waye et al., 1994; Patrinos et al., 2004; Shooter et al., 2015; Waye et al., 2017;).

Concerning the third family, in this paper we report for the first time a single nucleotide mutation involving the last nucleotide of the β RNA transcript, a transversion C > A in the position c.*132. This mutation was observed in a 28-year-old male patient in compound heterozygosity with *HBB*: c.118C > T. Given the patient’s hematological data we infer the possible pathological effect of this mutation. Additional evidence was provided by his son data, who is a simple heterozygote for this allele and presents microcytic hypochromic features with elevated HbA_2_ typical of a beta-thalassemia trait (Table I). This mutation involves the last nucleotide of the HBB transcript, located nearly 20 bp upstream the poly-A signal (https://www.ncbi.nlm.nih.gov/gene/3043) and has a possible disrupting effect on transcription and RNA maturation processes. To our knowledge, there is a report of another transition in the same position (*132C > T) associated with c.92+1G > A in a patient with β-thal intermedia phenotype (Bilgen *et al.*, 2013). Although the patient’s phenotype reveals a clear evidence of the pathological effect of this mutation, further transcription and mRNA analysis are required for establishing said effect. It has come to our interest the unusual elevated HbA_2_ in this family (I-1 and II-2). Usually, this particular feature is associated with β gene promoter deletions or single nucleotide mutations that involve CACCC, CCAAT or TATA elements (Thein 2018). In both of these patients, sequence analysis showed no mutations in *HBB* promoter.

*HBG2*:c.-211C > T polymorphism was observed in heterozygosity in three members of the first family with normal HbF (I-2, II-2 and II-5, Table 1), and two members of the second family with elevated HbF (I-2 and II-1, Table 1), therefore, the effect of this variant on the induction of γ-globin chains cannot be easily explained in these Mexican families.

Beta thalassemia intermedia is a widely variable disease, although the vast majority of mutations are associated to β globin gene, other genes can be involved. For this reason, it is important not to despise other genetic factors, such as other globin genes (α, γ or δ) or genetic variants located outside the globin gene clusters. Additional genomic approaches could clarify the etiology, as well as the clinical heterogeneity of this pathology.

## Conclusions

We report for the first time three Mexican families with beta thalassemia intermedia due to different molecular defects. Likewise, we report a new single nucleotide mutation (*HBB*: c.*132C > A) involving the last nucleotide of the β-globin chain transcript; as well for the first time in Mexican population, two possible new DNA rearrangements, an α cluster duplication that includes both α genes, and a partial β gene deletion. *HBB*: c.118C > T mutation is the most frequent β-thal allele in Mexican population (Rizo-de la Torre *et al.*, 2017), for this reason it is not surprising to find its association with other mutations as a main cause of β-thal intermedia. β-thal intermedia is supposed to be uncommon in our population, whereas β-thal carriers are rather frequent suggesting that this condition might be underdiagnosed therefore it is important to identify unusual or new variants that could lead to complex cases of hemoglobinopathies.
